# Unimproved source of drinking water and its associated factors: a spatial and multilevel analysis of Ethiopian demographic and health survey

**DOI:** 10.1186/s12889-023-16354-8

**Published:** 2023-07-31

**Authors:** Fantu Mamo Aragaw, Mehari Woldemariam Merid, Tsion Mulat Tebeje, Muluken Gizachew Erkihun, Amensisa Hailu Tesfaye

**Affiliations:** 1 Department of Epidemiology and Biostatistics, Institute of Public Health, College of Medicine and Health Sciences, University of Gondar, Gondar, Ethiopia; 2 Department of Epidemiology and Biostatistics, School of public health, College of Health Science and Medicine, Dilla University, Dilla, Ethiopia; 3 Department of Surgery, School of medicine, College of Medicine and Health Sciences, University of Gondar, Gondar, Ethiopia; 4 Department of Environmental and Occupational Health and Safety, Institute of Public Health, College of Medicine and Health Sciences, University of Gondar, Gondar, Ethiopia

**Keywords:** Ethiopia, Multilevel analysis, Spatial analysis, Unimproved drinking water source

## Abstract

**Background:**

Drinking water quality has been a major public health concern in lower and middle income countries where access to improved water supplies is limited. Ethiopia is thought to have one of the worst drinking water infrastructures in the world. This study aimed to assess the spatial variation and determinants of using unimproved sources of drinking water in Ethiopia using recent nationally representative data.

**Methods:**

A population-based cross-sectional study was employed with the recent EDHS data of 2019. A total of 8663 households were sampled using a stratified two-stage cluster sampling method. Kuldorff’s SaTScan version 9.6 software was used to generate spatial scan statistics. ArcGIS version 10.7 software was used to visualize the spatial patterns of unimproved drinking water sources. A multilevel multivariable mixed-effect logistic regression was used to identify factors associated with the use of an unimproved drinking water source. In the multivariable multilevel analysis, those variables with a p-value < 0.05 were considered to be significant predictors of using an unimproved source of drinking water.

**Result:**

Around 31% (95% CI: 30%, 32%) of the population in Ethiopia uses unimproved sources of drinking water with significant spatial variation across the country. Households aged 41–60 [AOR = 0.69; 95%CI; 0.53, 0.89] as compared to the households aged 10–25, households having middle wealth index [AOR = 0.48; 95%CI; 0.40, 0.59], and households having a rich wealth index [AOR = 0.31; 95%CI; 0.25, 0.39] as compared to the poor households, living in high community literacy level [AOR = 0.36; 95%CI; 0.16, 0.80], living in high-level community poverty [AOR = 3.03; 95%CI; 1.32, 6.98], rural residence [AOR = 7.88; 95%CI; 2.74, 22.67] were significant predictors of use of unimproved source of drinking water. Hot spot areas of use of unimproved drinking water sources were observed in Amhara, Afar, and Somalia regions and some parts of SNNPR and Oromia regions in Ethiopia. The primary clusters were found in Ethiopia’s Somalia and Oromia regions.

**Conclusion:**

Around one third of the Ethiopian population utilizes unimproved source of drinking water and it was distributed non-randomly across regions of Ethiopia. The age of the household head, wealth status of the household, residence, community poverty level, and community literacy level were found to be significantly associated with utilizing unimproved drinking water source. State authorities, non-governmental organizations and local health administrators should work to improve the quality of drinking water particularly for high risk groups such as communities living in high poverty and low literacy, poor households, rural residents, and hot spot areas to decrease the adverse consequences of using unimproved drinking water source.

## Background

Drinking water quality has been a significant public health issue, particularly in low and middle income countries where access to improved water supply and sanitation is limited [1, 2]. Access to safe drinking water is critical for the population’s health and overall well-being of the community [3]. Evidences suggest that unimproved drinking water and sanitation are responsible for around 50% of all diarrhoeal deaths worldwide [4]. The lack of basic Water, sanitation, and hygiene (WASH) services is thought to affect approximately 80% of people in low- and middle-income countries [4]. In Sub-Saharan Africa, particularly in Ethiopia, access to improved drinking water sources remains limited [5–7].

Universal access to safe drinking water is crucial for promoting the health and well-being of the population [3]. Utilizing unprotected water source increases the likelihood of various infectious diseases such as cholera, typhoid, dysentery, schistosomiasis, salmonellosis, and infections of the respiratory, skin, and eye systems [4, 8–10]. Also, when there is poor access to water, infections such as helminthiasis, scabies, and trachoma can occur [4, 11].

Safe drinking water is an essential element that is the fundamental requirement for sustaining life [4, 12–14]. The United Nations adopted Sustainable Development Goal in 2015, which calls for equitable access to safe and affordable drinking water for all by 2030 [2]. Access to improved water and sanitation is linked to lower morbidity [15–17], lower mortality, and a lower risk of diarrhea among children [15, 18].

In sub-Saharan Africa, WASH continues to be a significant public health concern with very low coverage [25]. Low-income households are the most vulnerable during periods of low water abundance [19]. Access to water and sanitation services remains significantly different between urban and rural areas, particularly in Sub-Saharan Africa [20].

The provision of WASH is a critical component of the prevention and control of communicable diseases [21]. WASH measures continue to be essential for reducing pathogen exposure that leads to water-borne illness, especially among children in low-resource settings [22–24]. Despite the fact that access to safe drinking water has yet to improve, the government of Ethiopia strives to ensure universal access to safe drinking water across populations in Ethiopia.The WASH project in Ethiopia has constructed well and spring water in both rural and urban settings to provide improved drinking water source for the population [32].

Ethiopia is thought to have one of the worst sanitation and drinking water infrastructures in the world [26]. In Ethiopia, access to improved water supply and sanitation has been very low and hence majority of the communicable diseases are associated with unsafe and inadequate water supply [10]. In accordance to the 2015 WHO Progress Report on Sanitation and Drinking Water, 43% of Ethiopia’s population relies on unimproved water sources [27].

In terms of access to clean drinking water, Ethiopia ranks among the lowest in Sub-Saharan Africa [28]. Moreover, evidence suggests that not every geographic area has equal access to safe drinking water [28–31]. Hence, this study aimed to explore the spatial pattern of unimproved source of drinking water and its associated factors in Ethiopia, using the recent nationally representative data. The findings of the study will help policymakers to implement measures to reduce the consequences of utilizing water from unimproved sources in Ethiopia.

## Methods

### Study design and setting

A repeated cross-sectional study design was conducted in Ethiopia using the intermediate EDHS 2019 which was carried out by the Central Statistical Agency in collaboration with the Federal Ministry of Health (FMoH) and the Ethiopian Public Health Institute. Ethiopia is the second most populous country in Africa, bordering Eritrea to the north, Djibouti, and Somalia to the east, Sudan and South Sudan to the west, and Kenya to the south.

### Data source

This study used the 2019 Ethiopian Mini Demographic and Health Survey (EMDHS) dataset, which was collected between March 21, 2019, and June 28, 2019. Data were obtained from the DHS website: www.dhsprogram.com by justifying the rationale for requesting the data and after obtaining an approval letter from the DHS. The study’s variables were extracted from the household Record (HR file) data set.

### Sampling procedures and populations

The EDHS employed a two-stage stratified cluster sampling procedure. Stratification was conducted by classifying the nine regional states and the two city administrations of Ethiopia, into urban and rural areas. Firstly, a total of 305 EAs (93 in urban, 212 in rural) were chosen independently with a probability proportional to each EAs. Second, from the newly formed household listing, a fixed number of 30 households/clusters were selected with an equal probability of systematic selection. The detailed sampling procedures are available on the measure DHS website in the 2019 EMDHS report (https://www.dhsprogram.com). We used the household record data set for this study with a total weighted sample of 8663 participants were included in this study.

### Study variables

The outcome variable for this study is the utilization of unimproved sources of drinking water. The drinking water source was classified as unimproved if a household gets drinking water from an unprotected dug well, unprotected spring, surface water, and others [7].

Individual and community-level independent variables were considered in this study. The age of the household head, education status of the household head, sex of the household head, household wealth, having television, and having a radio were some of the individual-level factors included in this study. Community-level factors, residence, community poverty level, community literacy level, and region were considered [33]. After checking their distribution, the community poverty and literacy levels were divided as high or low, and the median value was used as the cut-off point for classification since the data was not normally distributed. The community poverty level was classified as high if the proportion of households from the two lowest wealth quintiles in a given community was greater than the median value and low if the proportion was below the median value [34]. The community level literacy was the proportion of household heads in the community with at least a primary level of education, classified as high (proportion of households greater than median national value) whereas low (proportion of women below-median national value) [35]. In this study region was categorized into three categories; larger central [Tigray, Amhara, Oromia, and Sothern Nations Nationalities and Peoples Region], small peripherals [Afar, Somali, Benishangul, and Gambela], and metropolis [Harari, Dire Dawa, and Addis Ababa] based on their geopolitical features, which is consistent with a prior Ethiopian study [34, 36, 37].

### Data management and analysis

Before any statistical analysis, the data was cleaned, coded, and weighted using sampling weight for probability sampling and non-response to restore representativeness. We conducted a multilevel logistic regression analysis, assuming that each community has a different intercept and fixed coefficient, as well as within and between community variations, with a random effect applied at the cluster level. In the multivariable model, variables with p-values less than 0.2 in the bivariable analysis were fitted, and the Adjusted Odds Ratio (AOR) with a 95% CI and p-value of 0.05 were used to declare a significant association with the outcome. The goodness of fit was checked using deviance. The Variance Inflation Factor (VIF) was used to test for multicollinearity among the selected explanatory variables.

### Spatial analysis

Spatial analysis was conducted using the Geographic Information System (GIS) application to assess geographic variations of unimproved sources of drinking water cases among the 2019 EDHS clusters. We calculated the proportions of unimproved drinking water cases in each survey cluster, then appended the latitude and longitude coordinates of the selected EAS in the 2019 EDHS survey.

The spatial autocorrelation statistic (Global Moran’s I) was used to determine whether unimproved drinking water sources in Ethiopia were dispersed, clustered, or randomly distributed. Moran’s I index is the correlation coefficient, which measures the degree of association between a single variable with itself at different points in space as a function of the distance between points [38]. Furthermore, incremental autocorrelation was used to identify the distance band in which spatial processes promoting clustering were most prominent. Also, Getis-OrdGi* statistics were computed to determine how spatial autocorrelation differs across study locations by calculating GI* statistics. Statistical output with a high GI* implies a “hotspot,“ whereas a low GI* denotes a “cold spot” [39]. In addition, Z-score is calculated to identify significant hotspot and cold spot areas. The spatial interpolation technique was used to predict the unsampled/unmeasured value from sampled measurements. For unsampled cluster prediction, we employed a geostatistical ordinary Kriging spatial interpolation technique.

Bernoulli-based model spatial scan statistics were used to identify statistically significant clusters of unimproved drinking water sources among households. Households having unimproved drinking water sources were utilized as cases and those households who have access to improved drinking water sources as controls to fit the Bernoulli model. The most likely clusters were determined using p-values and likelihood ratio tests based on 999 Monte Carlo replications.

### Parameter estimation and mode selection

The random effects, which are measures of variation in the use of unimproved sources of drinking water across communities or clusters, were expressed in terms of the Intra-Class Correlation (ICC), the median odds ratio (MOR), and the proportional change in variance (PCV). The ICC shows the differences between clusters in the utilization of unimproved sources of drinking water among households. The ICC is calculated as ICC=$\frac{VA}{ VA+3.29}*100$, Where; VA represents the area-level variance [40–42]. The MOR indicates the central value of the odd ratio between the highest and the lowest risk regions when two clusters are chosen at random. The MOR is calculated as MOR=e0.95$\surd VA$, where VA donates the area level variance [43, 44].

The PCV measures the proportion of total observed individual variation that can be attributed to differences between clusters. The PCV is calculated as;$\frac{Vnull-VA}{Vnull}*100$, whereas; Vnull represents the variance of the initial model, while VA represents the variance of the model with more terms [43, 44].

In multilevel analysis, four models were fitted. The first was the null model containing no independent variables which were used to check the variability of use of unimproved drinking water source in the community. The second (Model I) hierarchical models contain individual-level variables whereas the third (Model II) contains community-level variables. In the fourth model (Model III) both individual and community-level variables were considered in the analysis simultaneously.

In the random effect analysis, the intra-class correlation (ICC) in the null model revealed that cluster variability accounted for 77% of the overall variability of the unimproved drinking water source. If we randomly select households from two different clusters, households in the cluster with a higher risk of using an unimproved source of drinking water had 8.64 times higher odds of using an unimproved source of drinking water than households in the cluster with a lower risk of using an unimproved source of drinking water. The PCV increases from 29% in model I to 42% in the third model containing both individual and community-level variables), implying that \model III best explains the variations of using an unimproved drinking water source. The third model had the lowest deviance (5953) and was thus defined to be the best-fit model. All variables had VIF values less than 10, and the final model’s mean VIF value was 2.01, indicating the absence of multi-collinearity (Table 1).

**Table 1:** Random effect (community level-clustering) and model selection in the use of unimproved source of drinking water among households in Ethiopia,2019

Random effect					
		Null model	Model I	Model II	Model III
** VA**		11.12	7.88	6.67	6.45
** ICC**		0.77	0.70	0 .67	0.66
** MOR**		8.64	7.24	6.67	6.55
** PCV**		Ref	0.29	0.40	0.42
**Model comparison**					
** Deviance**		6222	6031	6101	5953
** Mean VIF**		-	1.78	1.63	2.01

ICC = Inter cluster correlation coefficient, MOR = Median odds ratio, PCV = proportional change in variance

### Ethical consideration

All methods were carried out following relevant guidelines of the Demographic and Health Surveys (DHS) program. All experimental protocols were approved by the Institutional Review Board (IRB) of University of Gondar. Informed consent was waived from the International Review Board of Demographic and Health Surveys (DHS) program data archivists after the consent paper was submitted to the DHS Program, a letter of permission to download the dataset for this study. The dataset was not shared or passed on to other bodies and was anonymized to maintain its confidentiality. All methods were carried out in accordance with relevant guidelines and regulations.

## Result

A total weighted 8663 households were included in this study. Nearly half of the 4,120 participants (47.56%) have no formal education. More than three-quarters of all household heads (6,751 (77.93%)) were men. The vast majority (69.25%) of the participants in this study resided in rural areas. The magnitude of using an unimproved source of drinking water in Ethiopia was 31% (95% CI: 30%, 32%)( Table 2**)**.

**Table 2:** Socio-demographic characteristics of the study participants in Ethiopia, Ethiopian Demographic and Health Survey 2019 (n = 8663)

Variables	Categories	Source of drinking water		Total weighted frequency (%)
		Unimproved (%) n = 2,709 (31.28)	Improved (%) n = 5,953 (68.72)	
Age of women	10–25	295 (31.26)	650 (68.74)	946 (10.93)
	26–40	1,137 (32.04)	2,412 (67.96)	3,550 (40.99)
	41–60	841 (30.48)	1,919 (69.52)	2,760 (31.87)
	> 60	435 (30.95)	970 (69.05)	1,405 (16.22)
Education status of household head	No education	1,518 (36.86)	2,602 (63.14)	4,120 (47.56)
	Primary	897 (29.23)	2,172 (70.77)	3,069 (35.43)
	Secondary and higher	294 (19.95)	1,179 (80.05)	1,473 (17.0)
Sex of household head	Male	2,210 (32.74)	4,541 (67.26)	6,751 (77.93)
	Female	499 (26.11)	1,412 (73.89)	1,911 (22.07)
Have television	No	2,614 (36.28)	4,591 (63.72)	7,205 (83.17)
	Yes	96 (6.56)	1,362 (93.44)	1,457 (16.83)
Have radio	No	2,165 (34.63)	4,087 (65.37)	6,252 (72.17)
	Yes	545 (22.59)	1,866 (77.41)	2,410 (23.26)
Wealth index	Poor	1,658 (52.93)	1,474 (47.07)	3,132 (36.16)
	Middle	518 (30.93)	1,157 (69.07)	1,675 (19.34)
	Rich	533 (13.84)	3,321 (86.16)	3,854 (44.50)
Community level variables				
Residence	Urban	338(12.70)	2,325 (87.30)	2,663 (30.75)
	Rural	2,371(39.53)	3,627 (60.47)	5,999 (69.25)
Community poverty	Low	877 (17.77)	4,061 (82.23)	4,938 (57.01)
	High	1,832 (49.20)	1,891 (50.80)	3,724 (42.99)
Community literacy level	Low	1,766 (42.34)	2,405 (57.66)	4,172 (48.16)
	High	943 (21.01)	3,547 (78.99)	4,490 (51.84)
Region	Larger central	2,416 (31.92)	5,153 (68.08)	7,569 (87.38)
	Small peripherals	278 (43.82)	357 (56.18)	635 (7.34)
	Metropolis	15(3.25)	443 (96.75)	457 (5.29)

### Spatial analysis result

#### Spatial autocorrelation of unimproved source of drinking water

**Fig. 1:**
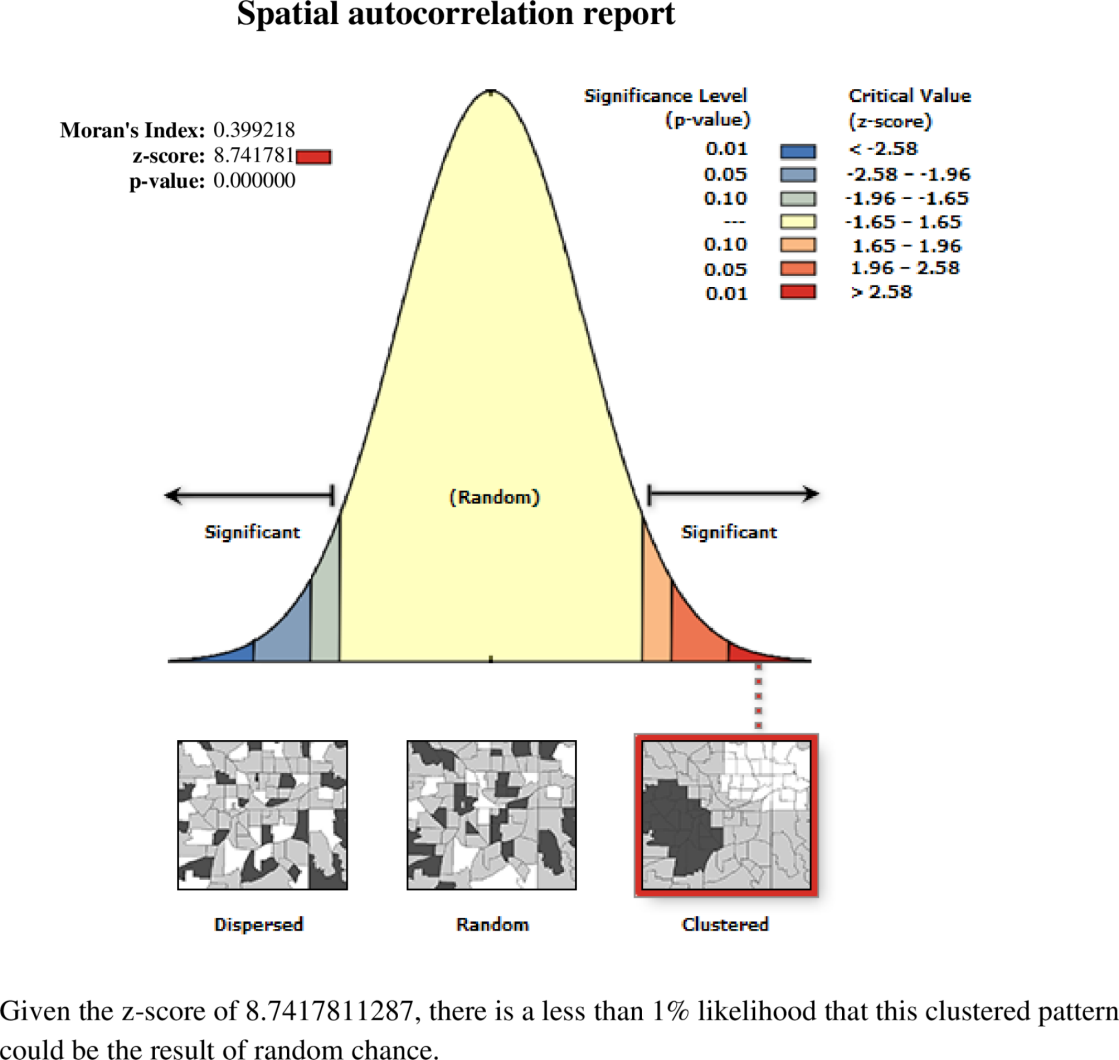
Spatial autocorrelation analysis of unimproved source of drinking water in Ethiopia, EDHS 2019

The spatial pattern of unimproved drinking water sources varied across Ethiopian regions. The result of the Global Moran’s Index value was 0.39 with a P < 0.001 and a Z score of 8.74 revealing that there was less than a 1% probability that this clustered pattern has been the result of a random chance (Fig. 1).

#### Spatial distribution of unimproved sources of drinking water

**Fig. 2:**
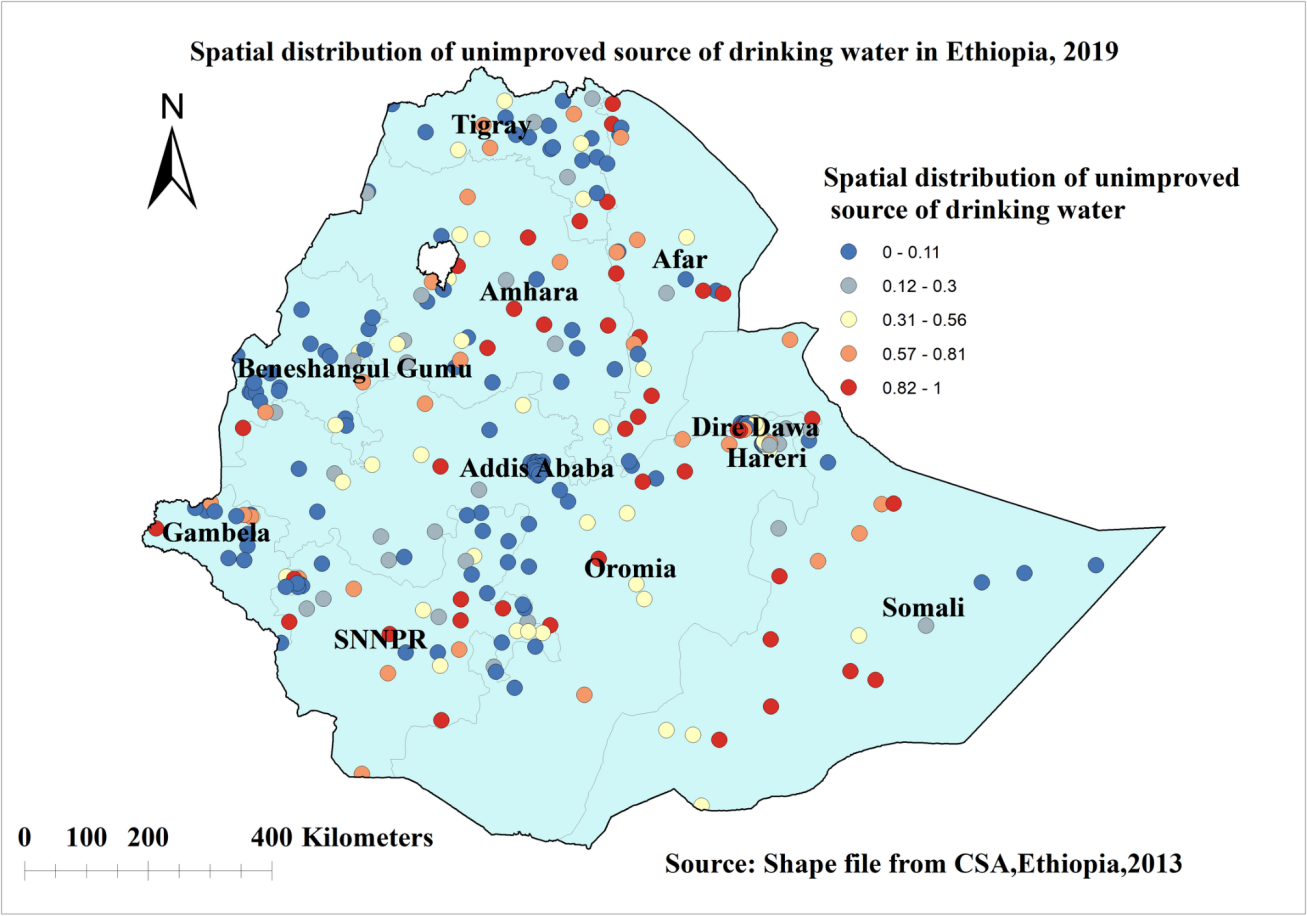
Spatial distribution of unimproved source of drinking water in Ethiopia, EDHS 2019

The highest proportion of unimproved drinking water sources in Ethiopia was detected in Amhara, Afar, Tigray, Diredawa, Somalia, and some parts of SNNPR, as well as border areas of Gambela and Benishangul gumuz regions. As indicated in Fig. 2 the red color represents highest prevalence of use of unimproved drinking water source ranging from a proportion of 0.8 to 1, while the blue colour indicates the lowest prevalence of use of unimproved drinking water source wasindicated by blue colour (Fig. 2).

#### Hot spot areas of unimproved sources of drinking water

**Fig. 3:**
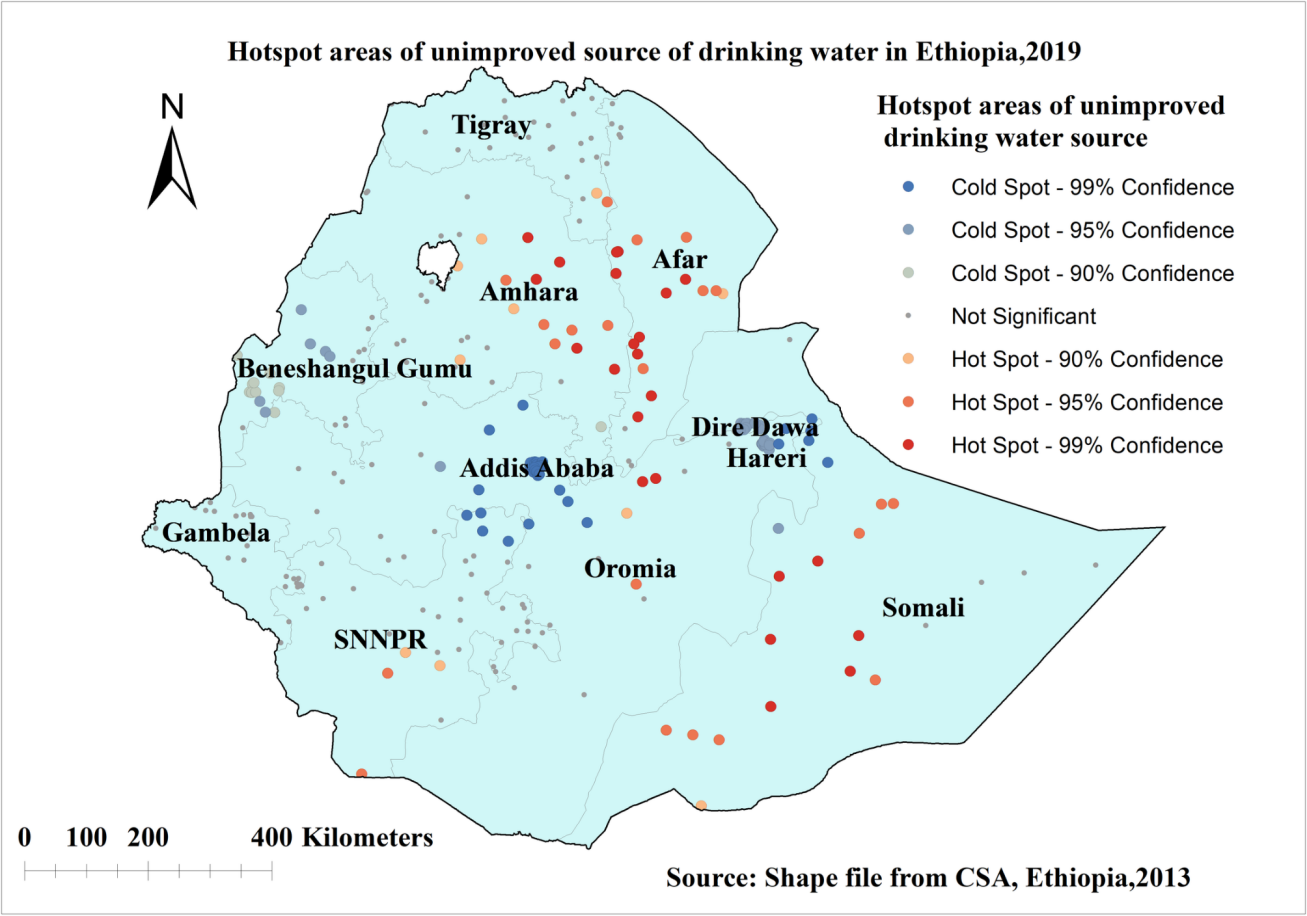
Hot spot analysis of unimproved source of drinking water in Ethiopia, EDHS 2019

Significant high proportion (hotspot) areas of unimproved sources of drinking water were indicated in red colour and they were observed in Amhara, Afar, Somalia regions and some parts of SNNPR and Oromia regions in Ethiopia. The low proportion (cold spot) areas of unimproved sources of drinking water were observed in Addis Ababa, Diredawa, Harari, and some part of Benishangul gumuz regions of Ethiopia as indicated in blue colour (Fig. 3).

#### Kriging interpolation of unimproved sources of drinking water

**Fig. 4:**
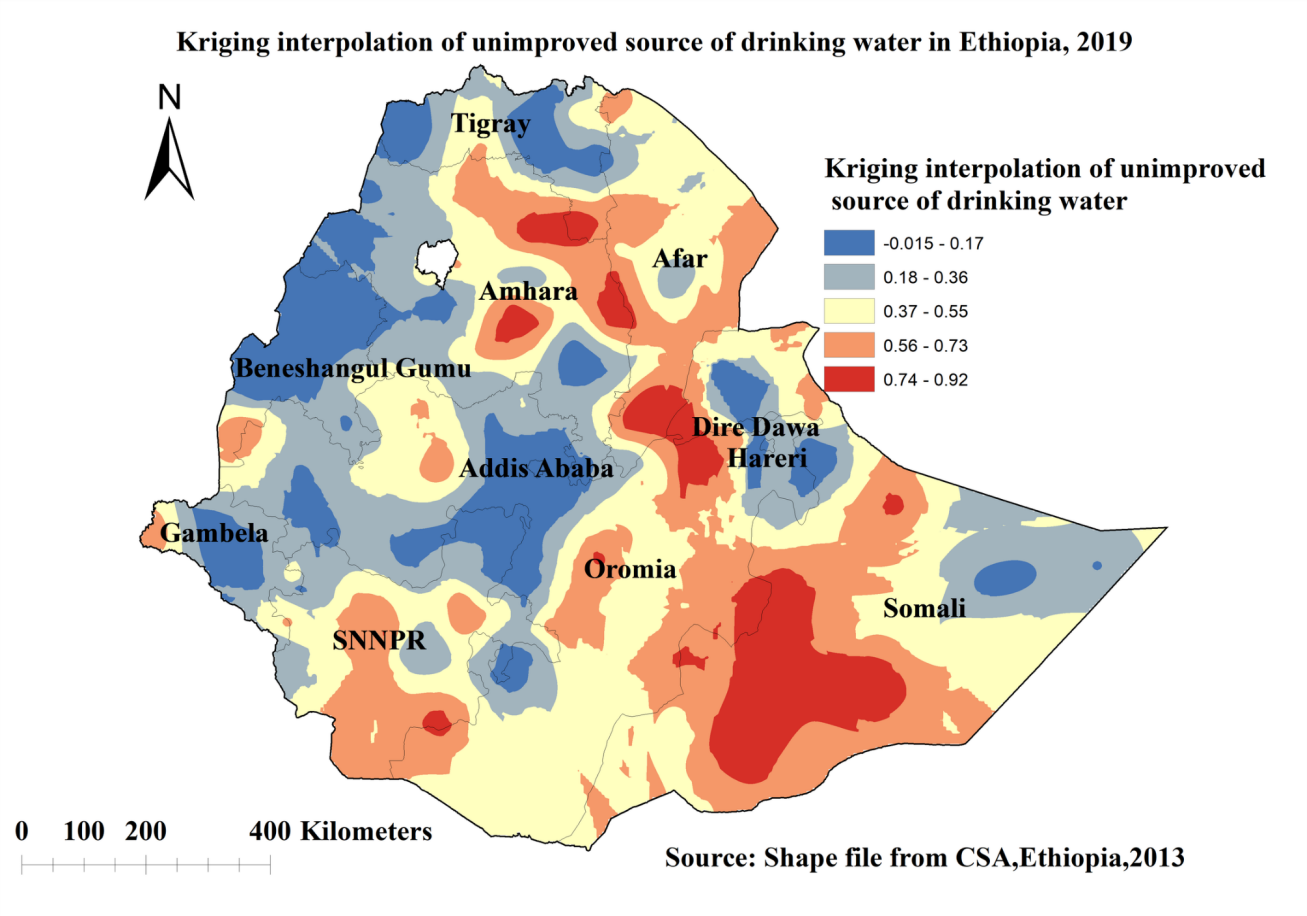
Kriging interpolation of unimproved source of drinking water in Ethiopia, EDHS 2019

The proportion of high-risk areas predicted for unimproved sources of drinking water was extremely high which range from 0.74 to 0.92 in Somalia, Amhara, Afar, and Dire Dawa regions which was indicated in red colour. Whereas the lower predicted unimproved source of drinking water was seen in Addis Ababa, Benishagul gumuz, Gambela, Tigray regions, and some parts of Dire Dawa, and Harari regions which was indicated in blue colour **(**Fig. 4**)**.

#### Spatial scan statistical analysis of unimproved source of drinking water

**Fig. 5:**
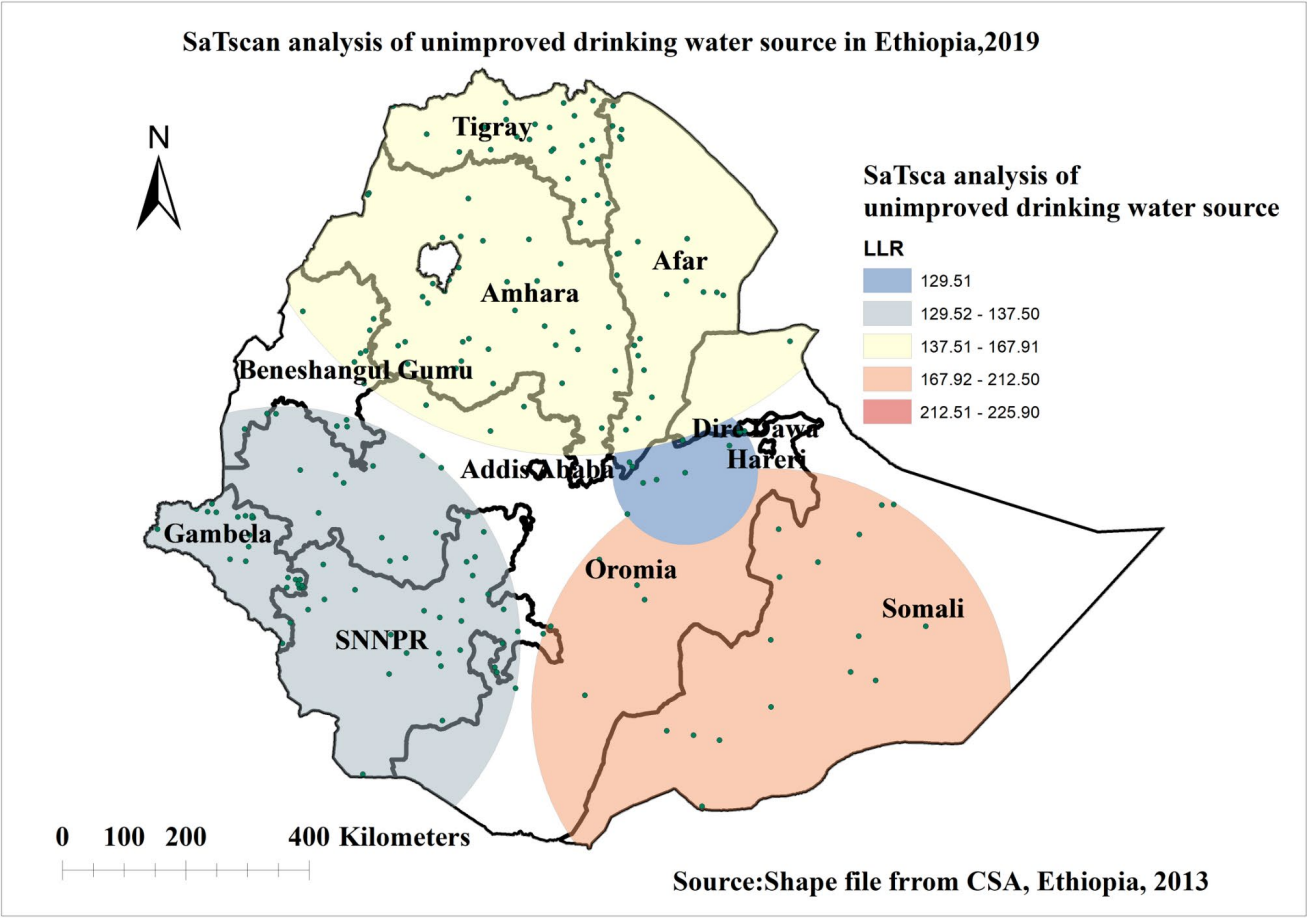
Spatial scan statistics analysis of unimproved source of drinking water in Ethiopia, EDHS 2019

A total of 197 significant clusters were identified of which 22 were most likely (primary) and 175 secondary clusters. The primary clusters were found in Ethiopia’s Somalia and Oromia regions. The primary clusters were centered at 5.479641 N, 42.196835E with a 387.42 km radius, a relative risk (RR) of 2.66, and a Log-Likelihood ratio (LLR) of 212.49, at p < 0.001. It showed that households living in the spatial widow had 2.66 times higher risk of using unimproved drinking water sources (Fig. 5).

#### Factors associated with using unimproved drinking water source

Based on the final model result, the age of the household head, wealth status of the household, residence, community poverty level, and community literacy level were found to be significantly associated with having an unimproved drinking water source. The odds of utilizing drinking water from unimproved sources among households aged 41–60 was lowered by 31%[AOR = 0.69; 95%CI; 0.53, 0.89] than households aged 10–25. The odds of using an unimproved source of drinking water for the middle and rich household index were decreased by 52% [AOR = 0.48; 95%CI; 0.40, 0.59],69%[AOR = 0.31; 95%CI; 0.25, 0.39] as compared to households having poor wealth index. The odds of utilizing water from an unimproved source among those households living in high-level community literacy was lower by 64% [AOR = 0.36; 95%CI; 0.16, 0.80] than women living in low community literacy levels. The odds of using an unimproved source of drinking water among those households living in a high community poverty level is 3.03 [AOR = 3.03; 95%CI; 1.32, 6.98] times higher than households living in a low community poverty level. Those households living in rural residences were 7.88 [AOR = 7.88; 95%CI; 2.74, 22.67] times high likely to use unimproved sources of drinking water than women living in urban areas **(**Table 3**).**

**Table 3:** Mixed effect analysis of factors associated with use of unimproved source of drinking water among households in Ethiopia, Ethiopian Demographic and Health Survey, 2019

Variables		categories	Null model	Model I		Model II	Model III
				AOR [95% CI]		AOR [95% CI]	AOR [95% CI]
Age of household head		10–25		1.00			1.00
		26–40		0.83 [0.65, 1.07]			0.82 [0.64, 1.05]
		41–60		0.70 [0.54, 0.91]			0.69 [0.53, 0.89]**
		> 60		0 0.77 [0.57, 1.03]			0.76 [0.56, 1.02]
Education status of household head		No education		1.00			1.00
		Primary		0 0.96 [0.81, 1.14]			0.98 [0.82, 1.16]
		Secondary and higher		0 0.87 [0.67, 1.12]			0.90 [0.69, 1.17]
Sex of household head		Male		1.00			1.00
		Female		0.84 [0.70, 1.01]			0.86 [0.71,1.04]
Have Radio		Yes		1.11 [0.93, 1.32]			1.10 [0.93, 1.32]
		No		1.00			1.00
Have Television		Yes		0.55 [0.38, 0.80]			0.74 [0.51, 1.07]
		No		1.00			1.00
Wealth index		Poor		1.00			1.00
		Middle		0.46 [0.38, 0.56]			0.48 [0.40, 0.59]***
		Rich		0.28 [0.22, 0.35]			0.31 [0.25, 0.39]***
**Community level variables**							
Residence	Rural				11.08[3.87, 31.72]		7.88[2.74, 22.67]***
	Urban				1.00		1.00
Community literacy level	Low				1.00		1.00
	High				0.33 [0.15, 0.74]		0.36 [0.16, 0.80]**
Community poverty	Low				1.00		1.00
	High				5.46 [2.37, 12.54]		3.03 [1.32, 6.98]**
Region	Large central				1.00		1.00
	Small peripheral				0.97 [0.43, 2.17]		0.88 [0.39, 1.97]
	Metropolitans				0.31 [0.08, 1.16]		0.35 [0.09, 1.28]

* = P-value < 0.05, ** = P-value < 0.01, *** = P-value < 0.001

AOR = adjusted odds ratio; CI = confidence interval

## Discussion

The purpose of this study was to assess the spatial variations and predictors of unimproved drinking water sources in Ethiopia. Around one third of the Ethiopian population utilizes unimproved source of drinking water. The finding was in line with a study done in Etiopia [45]. But it is lower than a study done in Eswatini [46], Zambia [47], and Ghana [48]. The disparity could be attributed to socioeconomic differences between countries as well as variations in the study period.

The spatial pattern of unimproved drinking water sources varied across Ethiopian regions. Significant hotspot areas of unimproved sources of drinking water were identified in Amhara, Afar, and Somalia regions and some parts of SNNPR and Oromia regions in Ethiopia. The finding is consistent with a study done in Ethiopia [49]. The primary clusters were found in Ethiopia’s Somalia and Oromia regions. Drought and scarcity of water in the Somali region make the population reliant on unimproved drinking water sources [29]. The majority of the population in Somali region is still dependent on seasonal water harvesting ponds, which are categorized as unimproved sources of drinking water. Most of the regions with high use of unimproved drinking water sources, such as the Afar and Somali regions, are inhabited by nomads and pastoralists who have difficulty accessing safe and adequate water supplies [50].

The odds of utilizing drinking water from unimproved sources among household heads aged 41–60 were lower as compared to household heads aged 10–25. The finding is consistent with a study done in Vietnam [51]. The possible reason might be those household heads aged 41–60 might be employed and will have a better chance to afford the cost of safe drinking water. Furthermore, old-aged household heads might be more aware of their well-being and might have a tendency to fulfill facilities that improve their health [48].

Households living within the rich wealth index were at low risk of getting their drinking water from unimproved sources as compared to households living within the poor wealth index. The finding is similar to a study done in Ghana [52, 53], Indonesia [54], and Bhutan [55]. The possible reason might be the rich can afford the cost of access to safe drinking water and the poor are disproportionately disadvantaged in the distribution of public utilities [45].

Households living in communities with high literacy are at low risk of getting their drinking water from unimproved sources as compared to households living in communities with low literacy. It was consistent with a study done in Ethiopia [29, 45, 56], Indonesia [54] Nigeria [31], Ghana [52], and low and middle-income countries [57]. The possible reason for this association is that the level of awareness about the health benefits of using a safe drinking water source is expected to rise as the community’s literacy level increases and more and the more educated community will have better access to safe drinking water infrastructure [58]. Another reason could be that households living with low community literacy know less about how to protect their available water source [29]. Households in rural areas are more likely to use their water from unimproved sources than households living in urban areas. The finding is consistent with a study done in Etiopia [29, 49, 59], and Nigeria [31]. People in rural areas typically have low socioeconomic status, which makes it difficult for them to obtain improved drinking water [45].

The study’s strengths were using a weighted nationally representative large dataset and an advanced model that took into account the data’s hierarchical structure. Furthermore, we conducted a spatial analysis and identified significant hotspot areas where unimproved drinking water sources were prevalent. As a limitation, the data did not include information about some predictor variables of an unimproved drinking water source since it was a secondary data analysis.

## Conclusion

Around one third of the Ethiopian population utilizes unimproved source of drinking water and it was distributed non-randomly across regions of Ethiopia. The age of the household head, wealth status of the household, residence, community poverty level, and community literacy level were found to be significantly associated with having an unimproved drinking water source. State authorities, non-governmental organizations and local health administrators should work to improve the quality of drinking water particularly for high risk groups such as communities living in high poverty and low literacy, poor households, rural residents, and hot spot areas to decrease the adverse consequences of using unimproved drinking water source.

## Data Availability

The mini Ethiopian Demographic and Health Survey 2019 data can be accessed from the Measure DHS program at www.dhsprogram.com.

## Abbreviations

AOR: Adjusted Odds Ratio
CI: Confidence Interval
COR: crude odds ratio
DHS: Demographic and Health Survey
EAs: Enumeration Areas
EMDHS: Ethiopian mini Demographic and Health Survey
GIS: Geographic Information System
GPS: Global positioning system
ICC: Intra Class Correlation
MOR: Median Odd Ratio
PCV: Proportional Change in Variance
VIF: Variance inflation factor
WHO: World Health Organization
